# Waist Training Corset: An Unusual Cause of Acute Lower Limb Ischemia

**DOI:** 10.7759/cureus.10465

**Published:** 2020-09-15

**Authors:** Max Murray Ramcharan, Adel Hanandeh, Brian Donaldson, Ali Safavi

**Affiliations:** 1 General Surgery, Columbia University College of Physicians and Surgeons, New York, USA; 2 Surgery, Harlem Hospital Center, New York, USA

**Keywords:** acute limb ischemia, thrombosis. medical education, corset, vascular surgery, peripheral vascular surgery

## Abstract

Acute limb ischemia (ALI) can occur due to many causes. This article illustrates a novel case of a very rare presentation and etiology of acute lower extremity ischemia. This case involves a middle-aged female with a history of smoking and obesity who presented with right lower extremity (RLE) pain. The patient had undergone a liposuction procedure a few days prior to her presentation and had been wearing a waist training corset. The patient was found to have multivessel thrombotic occlusive plaques starting from the right common iliac to the right tibial arteries. She was fully worked up and no other etiologies of her presentation was found. Thus, we concluded that her presentation was very likely precipitated by wearing the training corset, leading to right iliac artery thrombosis or perhaps a formal iliac atherosclerotic plaque destabilization and ipsilateral limb showering with athero-thrombi.

## Introduction

Acute limb ischemia (ALI) refers to a rapid decrease in lower limb blood flow due to acute occlusion of a peripheral artery or bypass graft. Clinical events that cause ALI include acute thrombosis of a limb artery or bypass graft, embolism from the heart, or a diseased artery, dissection, and trauma (from the severing of an artery or thrombosis) [1-2]. There is, however, a growing list of uncommon causes of ALI, which include aortic intimal sarcoma [3], popliteal artery entrapment syndrome [4], and prolonged arterial vasospasm of multiple vessels in multiple extremities simultaneously [5].

This case report discusses yet another unusual culprit behind ALI, a waist training corset resulting in ALI of the right lower extremity (RLE). We discuss the case of a 51-year-old female who presented with classic symptoms of ALI. Workup revealed occlusion of major arterial branches to the RLE, necessitating emergent surgical evacuation and stenting. This patient had recently undergone abdominal liposuction and was using a waist training corset adjusted tightly across the torso, which likely resulted in the occlusion of the arterial inflow, leading to thrombus formation and ALI.

## Case presentation

The patient was a 51-year-old female, with a past medical history significant for heavy smoking for many years and recent liposuction of the abdomen and gluteal regions, who presented to the ED with a complaint of severe pain in the RLE. She reported having undergone the liposuction procedure the day before her presentation and she had been wearing a tight waist training corset since. The patient denied any trauma to her RLE or any previous episodes of such symptoms. On presentation, she had a temperature of 97.6 °F, a heart rate of 103 bmp, blood pressure of 169/95 mmHg, and SaO_2_ of 100%.

On physical exam, the patient was noted to have an RLE tenderness to palpation in the right calf, and palpable but faint distal dorsalis pedis and posterior tibial pulses in the RLE. No deformities, edema, or any neurological deficits were noted. The patient endorsed the worsening of pain and increasing numbness in the RLE. Bloodwork at the time of admission was unremarkable; no significant abnormalities were noted.

**Figure 1:**
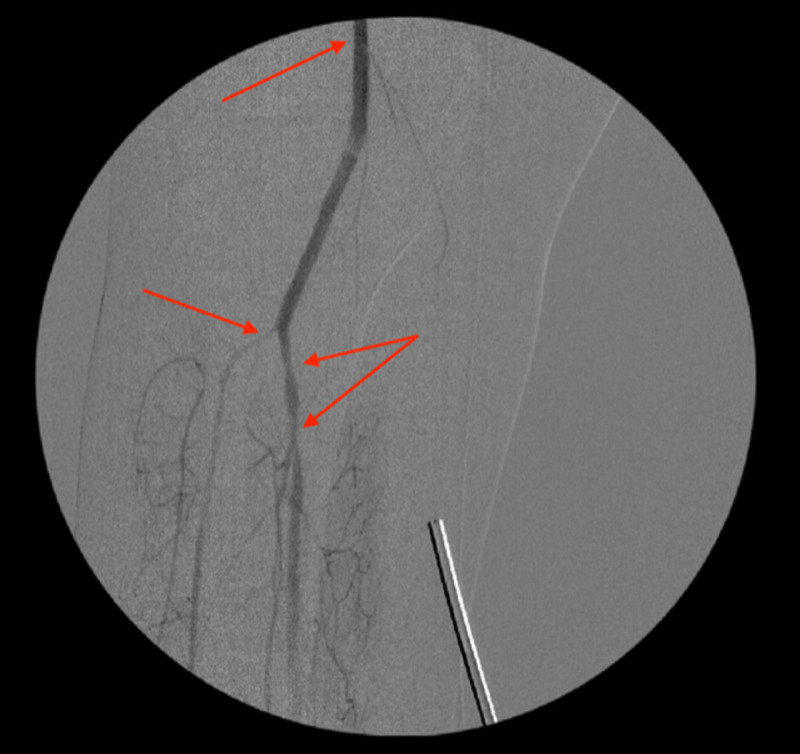
Angiogram after thromboembolectomy showing the right popliteal artery and right tibioperoneal trunk stenosis (arrows)

**Figure 2:**
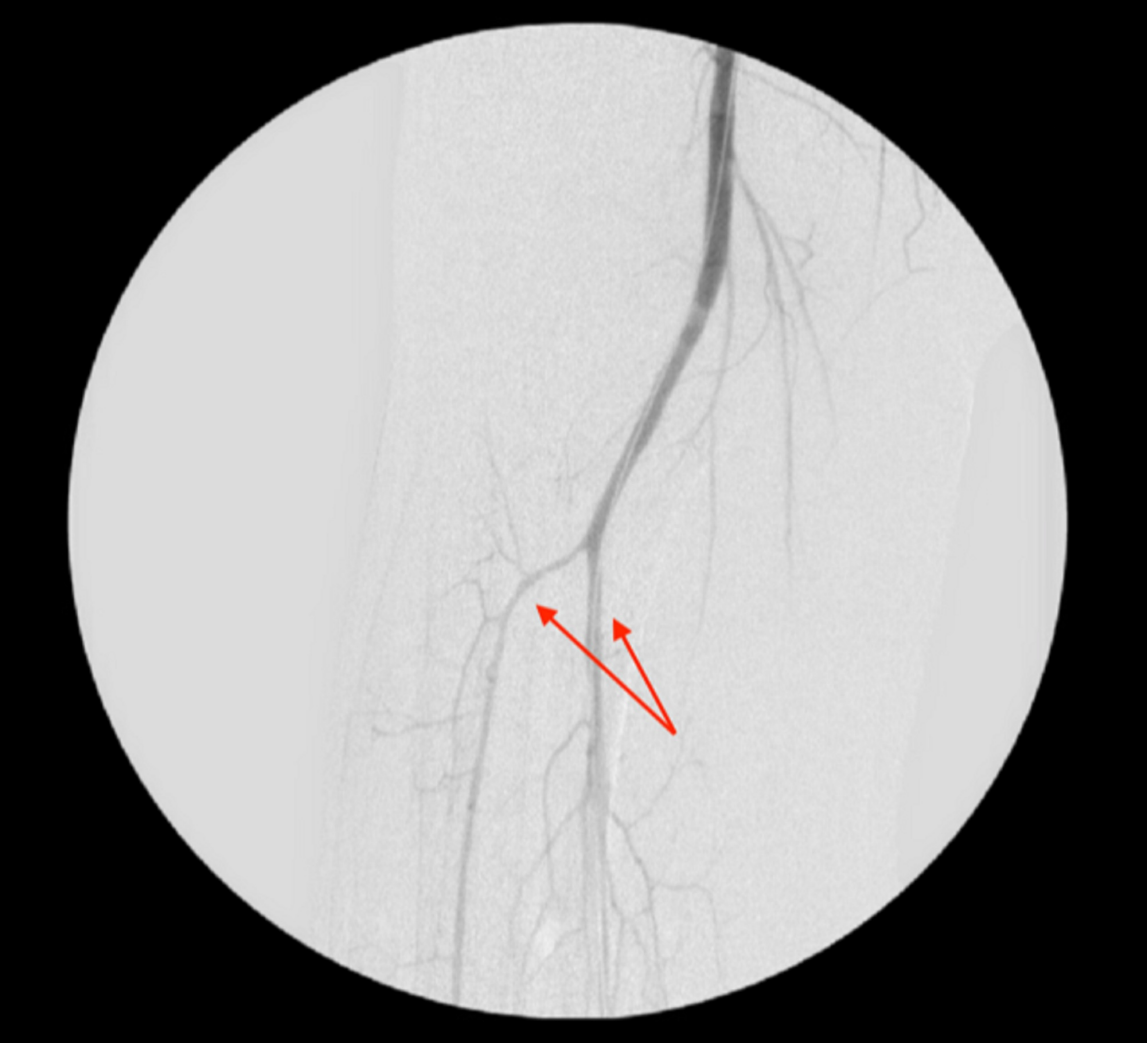
Angiogram indicating right tibioperoneal trunk after balloon angioplasty (arrows)

**Figure 3:**
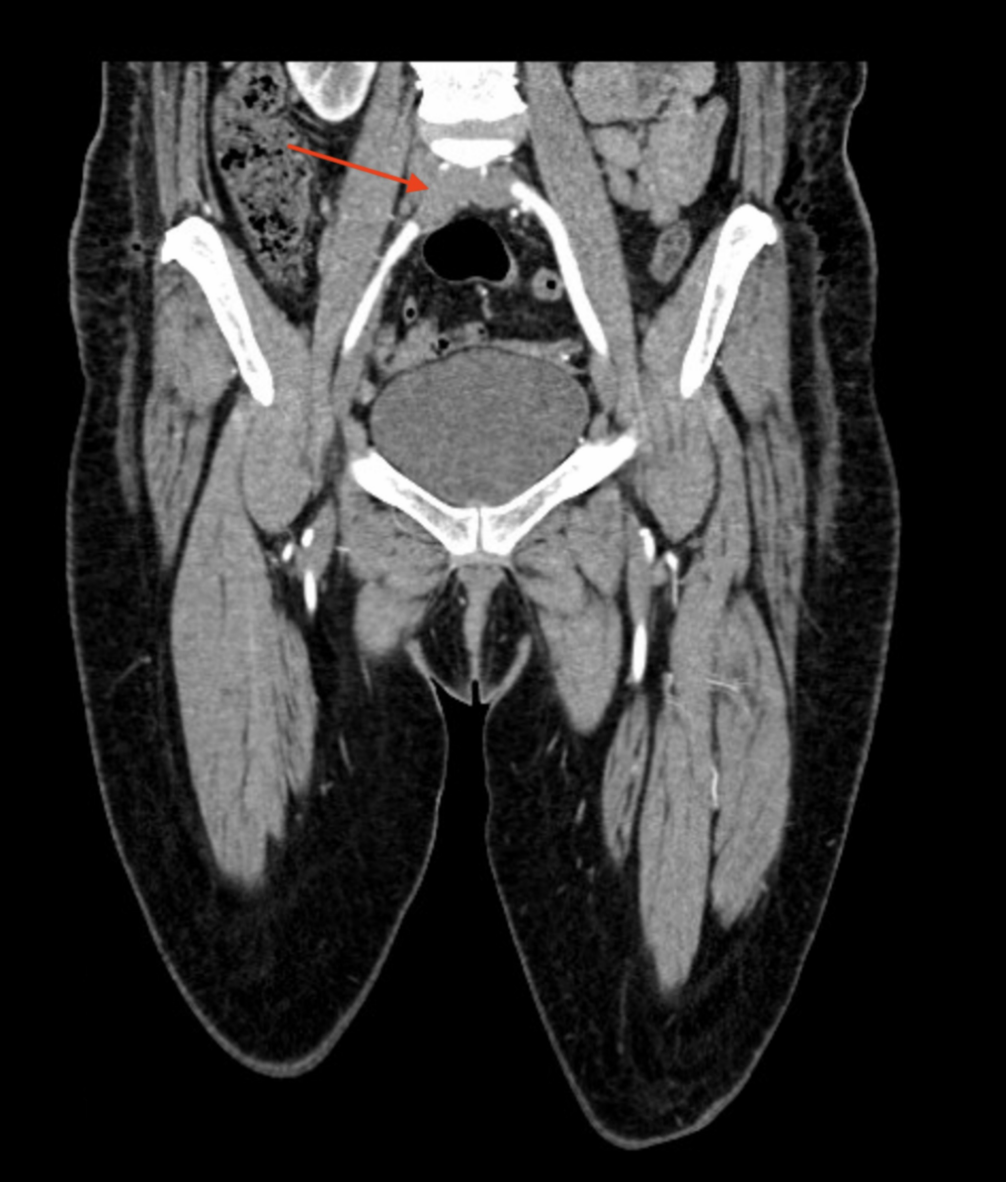
Computed tomography angiogram showing a right common iliac occlusion (arrow)

**Figure 4:**
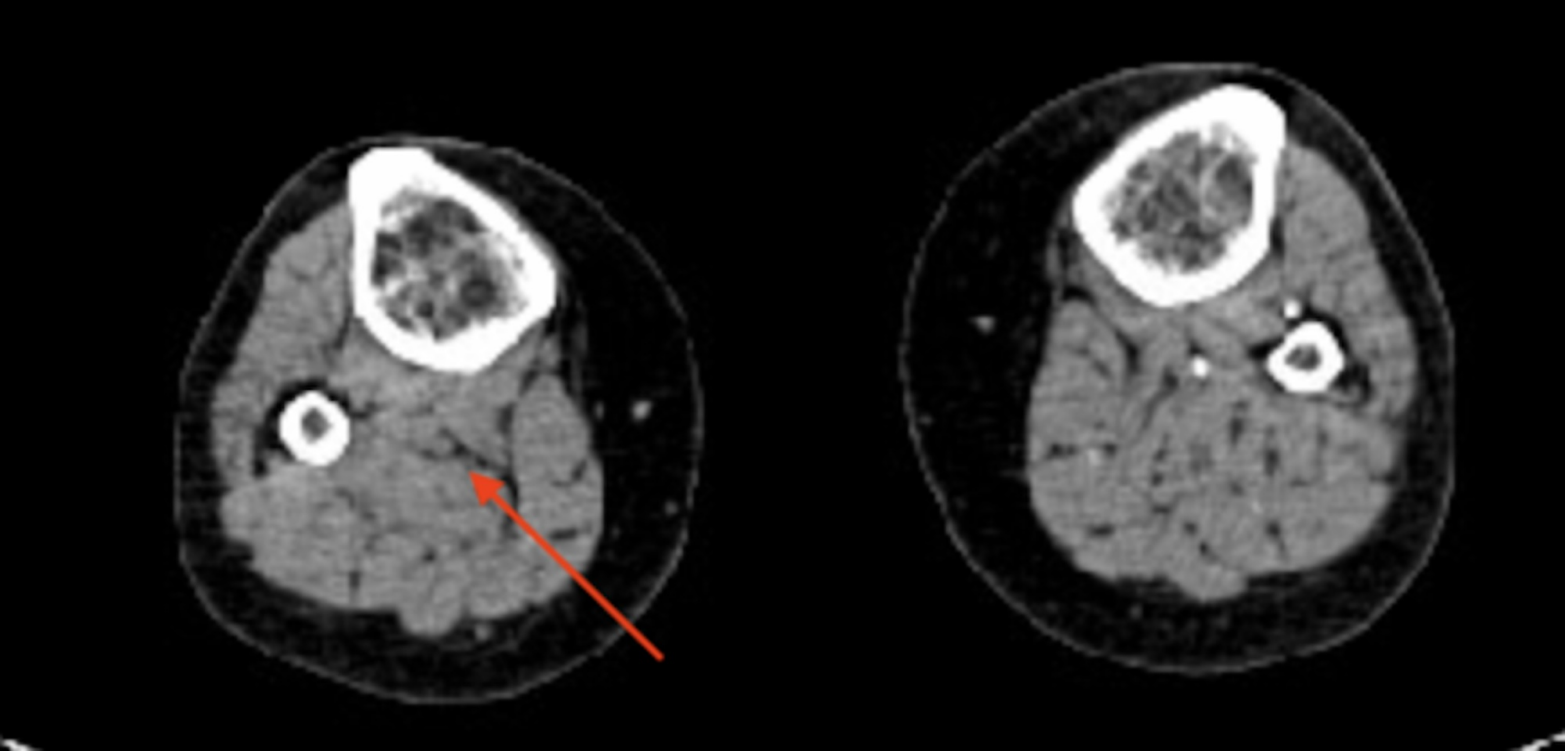
Computed tomography angiogram showing an occluded right popliteal artery (arrow)

A duplex of the RLE was performed and was negative for deep vein thrombosis (DVT). Computed tomography angiography (CTA) of the RLE indicated occlusive thrombus extending from the origin of the right common iliac artery into the proximal right external and internal iliac arteries, as well as a partially occluding thrombus in the distal portion of the right external iliac artery (Figure 1), thrombotic occlusion of the right distal popliteal artery (Figure 2), proximal occlusion of the right anterior tibial artery, and occlusion of the proximal posterior tibial artery with the reconstitution of flow (Figures 3,4). The patient was immediately placed on a heparin drip and was rushed to the operating room for emergent thromboembolectomy.

**Figure 5:**
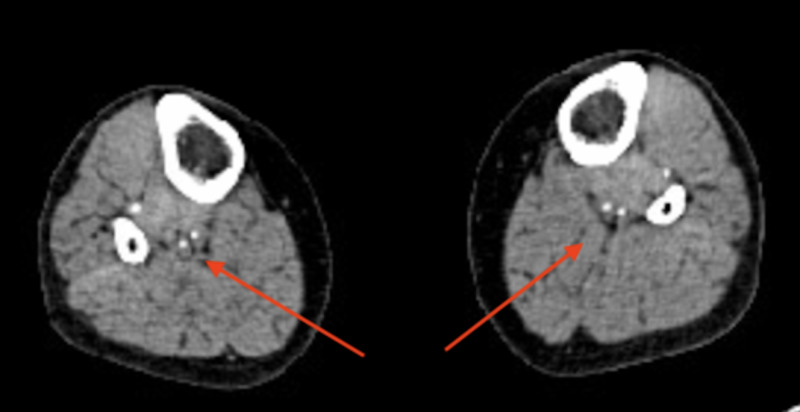
Computed tomography angiogram showing a reconstitution of flow in right anterior and posterior tibial arteries (arrows)

**Figure 6:**
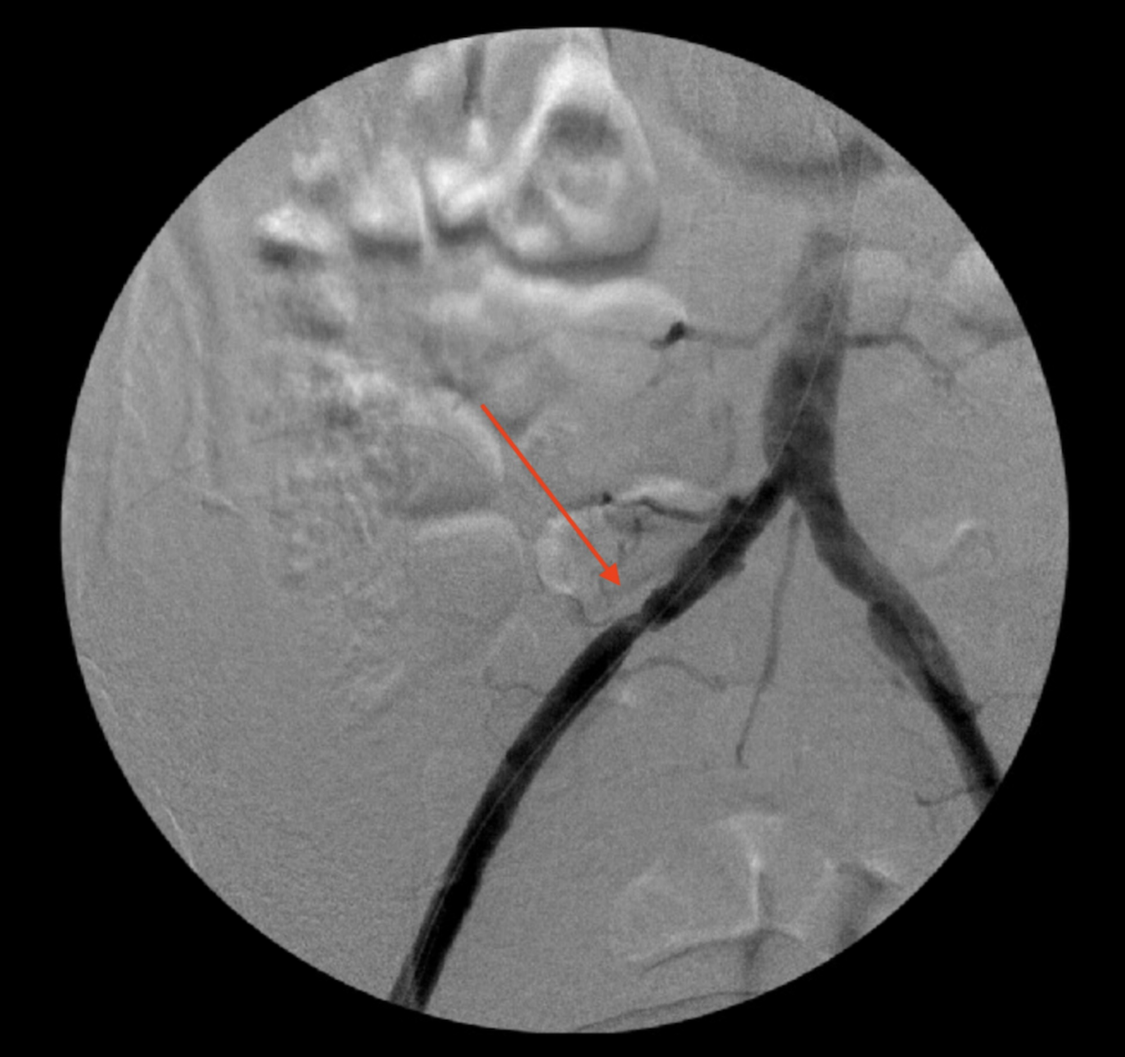
Angiogram showing right common iliac artery stenosis (arrow)

**Figure 7:**
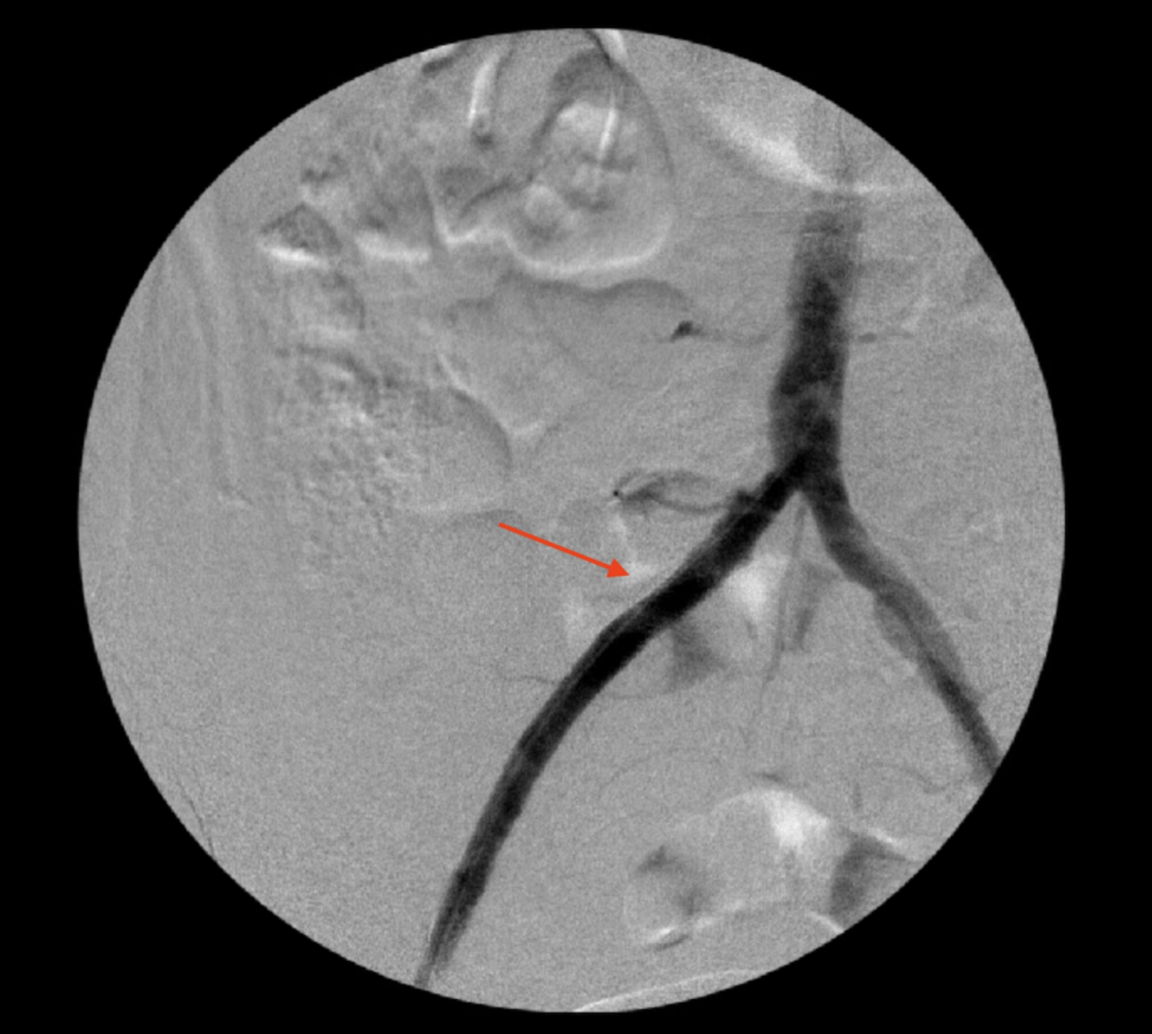
An angiogram showing right common iliac artery after thromboembolectomy and stent (arrow)

**Figure 8:**
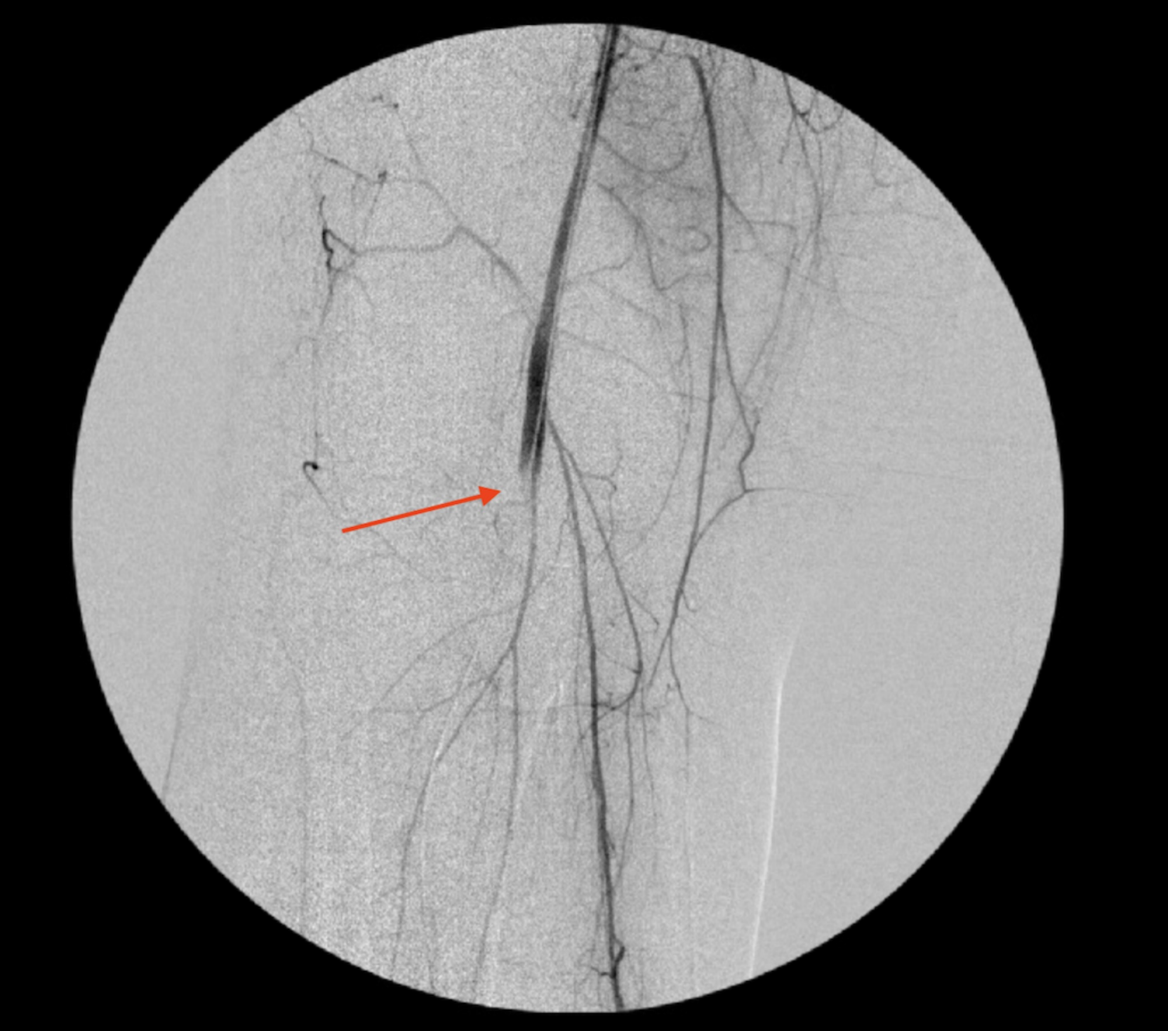
An angiogram showing right popliteal artery occlusion (arrow)

**Figure 9:**
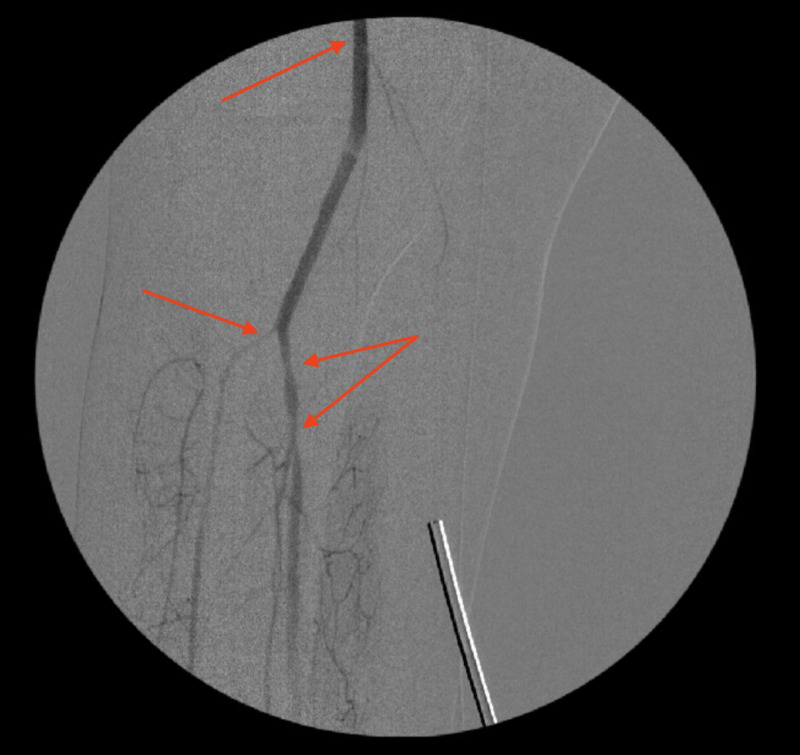
An angiogram showing right popliteal artery after thromboembolectomy, and right tibioperoneal trunk stenosis (arrows)

Intraoperatively, a cut-down incision was made in the right groin, and the right common femoral artery was isolated. Then, a longitudinal arteriotomy was done and the atheromatous plaque was identified and removed from the common femoral artery. Thereafter, a 7 French (7 Fr) Fogarty catheter was inserted through the common femoral artery proximally into the right common iliac and the thromboembolic clot was removed. The initial angiogram suggested a short stenotic segment in the right common iliac artery, and a size 9 F x 37 mm stent was placed, and the resolution of the common iliac stenotic area was confirmed with the completion angiogram (Figures 5,6). A 3 Fr Fogarty catheter was then passed distally and the thromboembolic clot in the popliteal artery was evacuated. The completion angiogram suggested patency of popliteal artery (Figures 7,8), small-caliber anterior tibial artery, and short-segment stenosis in the tibioperoneal trunk, which were corrected with 3 Fr x 55 mm balloon angioplasty and confirmed with post-angioplasty arteriogram (Figures 8,9), suggesting a return of flow past the tibioperoneal trunk allowing for reconstitution of flow to the right anterior tibial, right posterior tibial, and right peroneal arteries. A 2 mg alteplase was instilled via a catheter at the level of the distal femoral artery. Femoral arteriotomy, subcutaneous tissue, and skin were closed, and no complications were noted.

Postoperatively, the patient remained hemodynamically stable, and a repeat physical exam confirmed strong distal pulses in bilateral lower extremities, appropriately warm right leg, preserved neuromuscular function, and resolution in pain; she was able to ambulate without difficulty. A cardiac transthoracic echocardiogram was obtained, which showed no cardiac abnormalities or thrombus. The patient was kept on heparin drip postoperatively; she was discharged on postoperative day two on apixaban 5 mg twice daily for six months.

At multiple follow-up clinic appointments, the patient’s distal lower extremity pulses remained strong and palpable in the lower extremities throughout, extremities warm, and neuromotor function preserved. Ankle-brachial index at serial visits has indicated patent peripheral arteries in the bilateral lower extremities. The patient continues to deny pain, tingling, or symptoms suggestive of limb ischemia at follow-up visits. She was advised on smoking cessation, continued on appropriate anticoagulation, stressed the importance of continued surveillance, and asked to avoid the use of tight waist trainers in the future.

## Discussion

ALI is caused by many factors, including thrombotic, embolic, and other rare etiologies that may lead to a decrease in limb perfusion. In this case, the most likely explanation for the ALI was the waist training corset used after the recent abdominal liposuction and gluteal augmentation. Its position across the torso had likely created pressure against the pelvic structures and vasculature, precipitating a vascular injury leading to ALI. Also, wearing the training corset may have led to intimal injury and thrombus formation or a possible formal iliac atherosclerotic plaque destabilization resulting in an atherothrombotic embolization. This case represents a unique cause of ALI, with no similar etiologies leading to this pathology seen in our review of the literature.

Attention can also be drawn to the fact that this patient presented with unilateral acute lower limb ischemia despite the waist trainer presumably constricting vasculature bilaterally with approximately equal pressure. The exact reason for this remains unconfirmed but could be attributed to the patient’s previous smoking history affecting her vasculature asymmetrically and the presence of preexisting atherosclerotic plaque, thereby predisposing the diseased vessels to ischemic events when compared with others. It is likely that this patient had a preexisting iliac disease, but the application of the waist trainer may have resulted in atherosclerotic plaque rupture and acute thrombosis. Another possible explanation is that the right common iliac artery innately has a longer course than the left common iliac, due to the position of the inferior vena cava (IVC) to the right of the abdominal aorta. This longer course allows more opportunity for the right common iliac artery to become preferentially involved. In the absence of other obvious causative factors and the link between the development of this patient’s symptoms and the use of the waist training corset, it is logical to infer that this likely triggered the patient’s clinical picture.

Complications of liposuction procedures are well-documented in the surgical literature. In a literature review conducted in 2015 [6], many injuries related to liposuction are described, including abdominal wall injury, bowel perforation, hematoma, and venous thrombosis. There is also a previously existing case in which the right deep circumflex iliac artery, a branch of the right external iliac artery, is noted to have been injured during liposuction [7]. It is not unreasonable to question whether this procedure may have had a vascular complication, which may have contributed to her acute presentation a day postoperation. The patient described in this case report did not have any evidence of contrast extravasation from her lower extremity vessels, but this does not rule out the potential for superficial damage or resulting sequelae that could possibly result in intimal injury with further deleterious consequences after external compression with waist training corset.

The use of tissue plasminogen activator (TPA) is also an important area of discussion in this case as well as other cases of ALI. TPA is useful in maintaining the patency of vessels in select cases of ALI and can be administered in accordance with the American College of Cardiology/American Heart Association (ACC/AHA) 2005 Practice Guidelines for the Management of Patients With Peripheral Arterial Disease [8-10]. Studies suggest that thrombolysis is an effective and safe first-line treatment in cases of ALI, and the eligibility of patients as well as the correct infusion technique are essential elements for the success of this intervention [9-12]. The safety and efficacy of alteplase in particular have further been demonstrated by multiple studies advocating its use in this case [10,13-15].

## Conclusions

In this report, we sought to shed more light on an uncommon cause of an acute condition with a high rate of morbidity and mortality, requiring experience, high clinical suspicion, and specificity in questioning and tests in order for a timely diagnosis to be made. It is likely that external compression alone did not cause this presentation; rather, it may have resulted from a coupling of tight waist training corset and one or many of several preexisting conditions.

To the best of our knowledge, this case is one of the few cases to be reported about the condition we discussed. We strongly encourage educating our surgical community on factors that may precipitate vascular injury and plaque destabilization. Identification of high-risk patients is crucial prior to applying waist pressure that may contribute to such threatening pathologies.
